# The Association Between Perinatal Pharmacologic Treatments and Spontaneous Intestinal Perforation in Extremely Preterm Infants: A Propensity Score Matching Study

**DOI:** 10.3390/children12020142

**Published:** 2025-01-27

**Authors:** Wei-Hsin Cheng, Lo-Hsuan Tu, Ming-Chou Chiang, Yu-Ning Chen, Wei-Hung Wu, Kai-Hsiang Hsu

**Affiliations:** 1 School of Medicine, College of Medicine, Chang Gung University, Taoyuan 333, Taiwan; b0802016@cgu.edu.tw (W.-H.C.); b0802022@cgu.edu.tw (L.-H.T.); cmc123@cgmh.org.tw (M.-C.C.); eunice80221@cgmh.org.tw (Y.-N.C.); ch60819@cgmh.org.tw (W.-H.W.); 2 Division of Neonatology, Department of Pediatrics, Chang Gung Memorial Hospital, Taoyuan 333, Taiwan; 3 Graduate Institute of Clinical Medical Sciences, College of Medicine, Chang Gung University, Taoyuan 333, Taiwan

**Keywords:** spontaneous intestinal perforation, pharmacologic agents, hydrocortisone, inotrope, extremely preterm infants

## Abstract

**Background**: The impact of perinatal pharmacologic agents on spontaneous intestinal perforation (SIP) in extremely low-birthweight (ELBW, <1000 g) preterm infants remains inconclusive based on findings from retrospective cohort or case–control studies. This study aims to address this uncertainty by using propensity score matching (PSM) to reduce bias. **Methods**: We retrospectively reviewed ELBW infants in our unit between 2014 and 2023 to identify SIP cases. Confirmed through medical notes, surgical consultation, and author review, each SIP case was matched at a 1:3 ratio using propensity scores on factors including the gestational age (GA), birthweight, gender, and birth year. Pharmacologic agents commonly given antenatally and postnatally were analyzed. Only medications that were started 24 h before the onset of SIP or the corresponding age (PSM-controls) were included. **Results**: A total of 858 ELBW infants were reviewed, 28 SIP cases (GA 25.3 ± 2.1 weeks, BW 735 ± 167 g) were identified, and 84 PSM-controls were matched. The SIP cases received hydrocortisone (25% (7/28) vs. 9.5% (8/84), *p* = 0.037) and combined inotropic agents (17.9% (5/28) vs. 2.4% (2/84), *p* = 0.020) to a significantly greater extent. No differences were observed in the use of other medications. In logistic regression, the use of hydrocortisone and combined inotropes remained independent risks for SIP, with ORs (95% CIs) of 3.4 (1.1–10.9) and 2.1 (1.2–3.8), respectively. **Conclusions**: This first PSM-based study supported postnatal hydrocortisone and combined inotrope use as independent risks for SIP in ELBW infants. Clinicians should be aware of these risks and remain vigilant for SIP when administering hydrocortisone and inotropes.

## 1. Introduction

Spontaneous intestinal perforation (SIP) refers to an idiopathic condition that typically involves the distal ileum and occurs without focal signs of inflammation or necrosis in the intestinal tissue. SIP is a severe complication that is often observed in extremely low-birthweight (ELBW, birthweight <1000 g) preterm infants. Although emergent surgery may not always be necessary, the affected infants usually present with significant abdominal illness, delayed enteral feeding, respiratory distress, intra-abdominal infection, sepsis, or mortality [1]. Given the potential for SIP to become the most prevalent form of severe bowel disease in ELBW infants [2], despite its unclear pathophysiology, there is a need to identify those at risk for this condition. Several perinatal factors are linked to SIP, including lower gestational age (GA), decreased birthweight, and male sex; however, these factors remain nonspecific.

In addition to demographic factors, various pharmacologic agents have been studied, but no conclusive results have been obtained. Recent studies to identify treatment-associated risk factors that include SIP as the primary outcome are summarized in Table 1 [3,4,5,6,7,8,9,10,11,12,13,14,15,16]. For example, antenatal steroids (ANS), which are the standard care provided to stimulate fetal lung maturation, showed no significant difference in most studies, except for one study that reported an increased risk of SIP when ANS is used in combination with postnatal prophylactic indomethacin [13]. Magnesium sulfate (MgSO_4_) is given for neuroprotection, but concerns have been raised about the potential effects on intestinal motility and atony [7,16]. Indomethacin is a tocolytic agent that has been associated with intestinal hypoperfusion [15,16]. However, no definite conclusions regarding prenatal medication and the development of SIP have been reached.

The associations between SIP and postnatal pharmacologic regimens have been widely discussed. Postnatal steroids, including hydrocortisone and dexamethasone, are applied in extremely preterm infants to prevent bronchopulmonary dysplasia. Although these therapies target the lungs, they also affect other organ systems, including ileal crypt cells, which can result in alterations in the intestinal mucosal alignment and motility [3]. Some studies have suggested a possible link between early systemic steroid use and a greater risk of SIP [3,4,6,8,12,16]. Patent ductus arteriosus (PDA) is prevalent among ELBW infants, and pharmacologic interventions are typically employed to close the ductus. Prostaglandin inhibitors, such as indomethacin (in short supply in Taiwan since 2010) and ibuprofen, are traditionally the drugs of choice; both have been linked to SIP in retrospective studies. Whereas more than half of all studies suggested that indomethacin is a risk factor [4,5,6,8,10,14,16], only one of the three previously referenced studies reported an association [8]. An emerging agent for PDA, acetaminophen (paracetamol) has not yet been studied in relation to SIP. Systemic hypotension and intestinal hypoperfusion, which are often managed with inotropes (dopamine, dobutamine, or epinephrine), have also been found to be associated with SIP [5,11,12].

As demonstrated in Table 1, the associations between these medications and SIP remain inconclusive. A significant limitation of previous studies is their designs. All existing reports in which SIP is the primary outcome have been either retrospective cohort studies or matched case-control studies, making them susceptible to selection bias. Propensity score matching (PSM) is a statistical method that minimizes bias related to patient characteristics. It helps investigators to reduce the likelihood of confounding when analyzing retrospective nonrandomized observational data [17]. Consequently, this approach provides a more robust estimate of treatment effects. In this study, we aimed to utilize PSM to reduce confounding bias in the examination of pharmacologic risk factors for SIP in ELBW preterm infants.

## 2. Materials and Methods

### 2.1. SIP Cases

This study was conducted in the neonatal unit at Chang Gung Memorial Hospital, Linkou, a referral center in northern Taiwan with 50 intensive care beds and 54 sick-baby nursery beds. We reviewed the unit’s database between January 2014 and December 2023 to identify ELBW preterm infants and locate SIP cases based on the following criteria: diagnosis documented by the attending physician, consultation sheet from the surgeon, and verification by the authors through medical record review. Each SIP case was confirmed by evidence of an abrupt onset of radiographic pneumoperitoneum, along with physical signs of acute abdominal distension or bluish-black discoloration. Infants with suspected necrotizing enterocolitis, meconium ileus, or gastrointestinal malformations were excluded.

### 2.2. Propensity Score Matching

Using the cohort of ELBW preterm infants from the same period, the propensity scores for SIP were calculated based on a logistic regression model, incorporating variables including GA, birthweight, gender, and birth year. A greedy PSM strategy was employed to find cases with the closest estimated propensity scores, utilizing either nearest neighbor matching or nearest neighbor within caliper ≤0.2. Each SIP case was matched with 3 infants (1:3) to create a PSM-control group, ensuring an absolute standardized mean difference (ASMD) of less than 0.1.

### 2.3. Risk Factors

In addition to examining individual demographics, maternal history and conditions that may relate to neonatal stress, we focused on analyzing pharmacologic agents that were applied antenatally and postnatally. The antenatal medications of interest included ANS to promote fetal lung maturation, MgSO_4_ for fetal neuroprotection, and indomethacin as a tocolytic agent. Postnatal pharmacologic agents of interest included surfactants for treating respiratory distress syndrome, dexamethasone and hydrocortisone for the prevention of bronchopulmonary dysplasia (BPD), ibuprofen (intravenous or oral) and acetaminophen (oral) for ductus arteriosus closure, caffeine for apnea of prematurity, milrinone to treat pulmonary hypertension and inotropes (dopamine, dobutamine, and epinephrine) to treat hypotension (defined as the mean blood pressure less than postmenstrual age (PMA) in weeks). Our unit typically followed a stepwise protocol for inotropes: dopamine as the first-line treatment, dobutamine as the second-line, and epinephrine as the third-line for severe cases, often in combination with dopamine and dobutamine. Only the medications that given at least 24 h before the onset of SIP or the corresponding age (PSM-controls) were adopted.

### 2.4. Statistics

PSM was performed using SAS version 9.4 (SAS Institute, Cary, NC, USA), whereas the remaining statistical analyses were conducted via SPSS version 24 (IBM, Armonk, NY, USA). ASMD was used to compare SIP cases with the cohort and PSM-controls. Continuous variables were analyzed using independent-samples t tests, whereas categorical variables were analyzed via the χ^2^ test or Fisher’s exact test. Significant risk factors identified between SIP cases and PSM-controls were subsequently analyzed via multivariable logistic regression, with the results expressed as odds ratios (ORs) and 95% confidence intervals (CIs). A *p* value < 0.05 was considered statistically significant.

## 3. Results

Over a 10-year study period, 858 ELBW infants (mean ± SD GA 26.2 ± 2.2, BW 770 ± 147) were admitted to our unit. Among the cohort, 28 SIP cases were identified, yielding an annual incidence ranging 1.3% to 6.1% and an overall prevalence rate of 3.3%. The GA and BW of the SIP cases were 25.3 ± 2.1 weeks and 735 ± 167 g, respectively, which were significantly more immature than the cohort (GA *p* = 0.043). Nineteen (67.9%) of these 28 patients were male. The diagnosis of SIP was made at an average chronological age of 8.5 ± 4.7 days or a PMA of 26.5 ± 2.0 weeks. Of them, four infants developed SIP early (age 0–3 days), and they were more mature than infants with SIP after the fourth day of life (GA 27.8 ± 2.5 vs. 24.9 ± 1.7 weeks, *p* = 0.008). No difference was seen in other demographic characteristics or risk factors.

**Figure 1:**
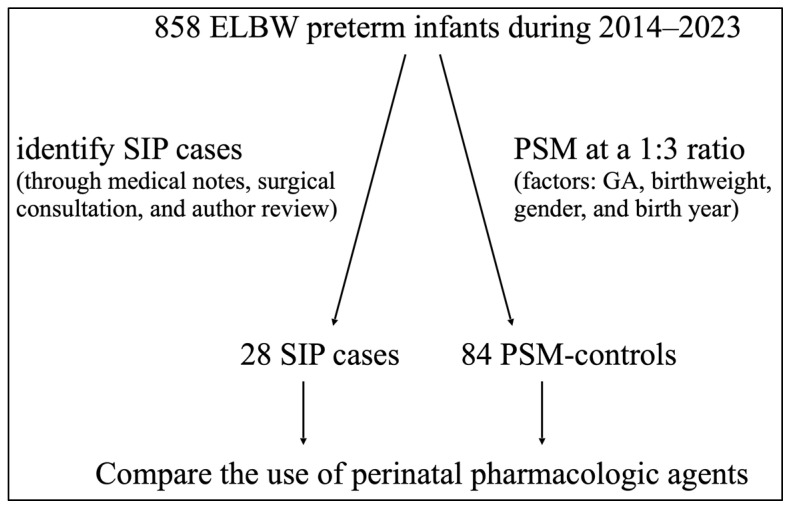
Enrollment flow diagram. ELBW: extremely low birthweight; SIP: spontaneous intestinal perforation; PSM: propensity score matching; GA: gestational age.

After PSM, 84 preterm infants were selected for the control group. The characteristics of the SIP cases, the cohort, and PSM-controls are presented in Table 2, which shows minimal mean differences between the SIP cases and PSM-controls. The enrollment flow diagram is illustrated in Figure 1.

The comparisons between SIP cases and PSM-controls are presented in Table 3. There were no significant differences in demographics or maternal conditions between the two groups, though fewer infants in the SIP group initiating feeding. However, there was a significantly greater usage of postnatal hydrocortisone to prevent BPD in the SIP group (25% (7/28) vs. 9.5% (8/84), *p* = 0.037). The SIP group also used more inotropic agents. Specifically, compared with PSM-controls (17.9% (5/28) vs. 2.4% (2/84); *p* = 0.016), a higher number of infants in the SIP group received a combination of three inotropes (dopamine + dobutamine + epinephrine). No significant differences were observed for other prenatal or postnatal pharmacologic agents. Furthermore, among infants who remained nil per os (NPO), the SIP group demonstrated higher usage of either hydrocortisone or inotropic agents. In multivariable logistic regression involving the use of hydrocortisone and combined inotropic agents, both risk factors remained independent for SIP, with ORs (95% CIs) of 3.4 (1.1–10.9) and 2.1 (1.2–3.8), respectively.

## 4. Discussion

This study explored the associations between multiple pharmacologic treatments commonly used in ELBW preterm infants and the risk of SIP. The results showed that postnatal hydrocortisone and combined inotropic agents (dopamine, dobutamine, and epinephrine) were independent risk factors for SIP. The strength of this study lies in the use of PSM, which reduced the selection bias compared with previous cohort or matched case-control studies and provided a more reliable estimation of the relationship between pharmacologic treatments and SIP.

In our 10-year single center cohort, the overall prevalence of SIP in ELBW preterm infants was 3.3%, consistent with findings from a recent national cohort [1]. Subgroup analysis based on the onset age of SIP showed that infants who developed SIP at 0–3 days of life were more mature compared to those with a later onset. This trend in GA differences has also been observed in a large-scale dataset [18]. However, we found no significant differences in other patient characteristics or perinatal risk factors, as noted in a previous study. This finding may be limited by the small number of early-onset cases in our study. Although fewer infants in the SIP group initiating feeding, similar to the findings of Rayyan’s study [11], both our study and Rayyan et al. suggested that the lack of feeding most likely reflects the severity of illness, particularly since both hydrocortisone and inotropic agents remained risk factors among neonates who were NPO.

This study revealed a significant association between postnatal hydrocortisone and a 3.4-fold increase in the risk of SIP. Our findings align with those of several studies that identified hydrocortisone as an independent risk factor for SIP, when SIP was used as the primary outcome measure [4,6,8,12,16]. In these studies, the use of postnatal hydrocortisone among SIP cases was reported from 7.3% to 31%. Although there were three other studies with different results [5,11,15], they had a relatively small number of SIP cases and may have had insufficient statistic power to disclose the association. In other large-scale prospective studies focused on preventing BPD in ELBW preterm infants, hydrocortisone-treated infants presented a higher incidence of gastrointestinal perforation, especially when the medication was used in combination with indomethacin [19]. Another individual patient data meta-analysis also showed that hydrocortisone combined with indomethacin increased the risk of SIP [20]. In a meta-analysis of randomized control trials that discussed the safety of systemic hydrocortisone for BPD prevention, a higher risk of bowel perforation was observed in infants who received hydrocortisone within the first postnatal week [21]. Because indomethacin was not available in Taiwan during this period, no infants received hydrocortisone in combination with postnatal indomethacin. However, we did not observe differences when hydrocortisone was used in combination with either intravenous or oral ibuprofen.

The proposed mechanism suggests that hydrocortisone affects insulin-like growth factors (IGFs), epidermal growth factor (EGF), transforming growth factor-alpha (TGF-α), and nitric oxide (NO) synthases [22,23]. In brief, steroids decrease the amount of IGFs in mesenchymal tissue and redistribute them to the villus and mucosa; they also deplete neuronal NO synthases, which influence the innervation of intestinal smooth muscle [22] and lower the level of TGF-α, an antiapoptotic factor, in the muscularis externa in relation to the inhibition of ileal smooth muscle proliferation [23]. These changes result in increased proliferation of the intestinal mucosa while compromising the submucosal thickness, potentially predisposing infants to SIP.

The study also revealed a significant association between the use of inotropes in combination (dopamine + dobutamine + epinephrine) and a 2.1-fold increased risk of SIP. Although previous studies showed an association between dopamine alone and SIP, our results indicated that inotropes used in combination were an independent risk factor for SIP. The possible mechanism involves systemic hypotension or epinephrine-related intestinal hypoperfusion and subsequent ischemia-reperfusion injury associated with SIP. Epinephrine is an endogenous catecholamine with β-1 and nonselective α effects and serves as both a potent inotrope to increase cardiac output and a vasopressor to increase systemic vascular resistance (SVR). In cases of hypotension and hypoperfusion, blood flow to the intestine is often compromised, increasing the vulnerability of intestinal circulation. Given that epinephrine is usually reserved for severe hypotension and is used in combination with other inotropes, our findings may reflect patients’ deteriorating conditions.

We must also consider the effect of vascular constriction caused by epinephrine. In one animal experiment, although systemic blood flow increased with epinephrine, it appeared to divert blood away from the gut, thereby decreasing intestinal microcirculation [24]. When hemodynamics improve, these initially low-perfusion areas may experience ischemia-reperfusion injury due to the induction of reactive oxygen species and proinflammatory factors, which negatively affect gut integrity.

Other perinatal medications that are commonly discussed were not associated with SIP in our study. No antenatal medication (ANS, indomethacin, or MgSO_4_) was significant in our study, which is consistent with those of the majority of previous studies. The lack of significance of postnatal dexamethasone corresponds with findings from other studies [4,11,12]. However, we have to cautiously interpret it, as only four infants in our cohort received postnatal dexamethasone. We did not find either intravenous or oral ibuprofen to be a risk factor, and the finding is consistent with those of a recent systematic review [25]. This study is the first to investigate whether acetaminophen is associated with SIP, and our negative finding aligns with those of another systematic review on the safety of acetaminophen for PDA in preterm infants [26]. However, there were only three such cases in both groups; hence, a large-scale study is warranted. Our study is also the first to clarify that there was no association of milrinone with SIP. Surfactant and caffeine were not associated with SIP.

This study’s limitations include the retrospective nature of the data collection, which may have led to missing or incomplete information. Additionally, the lack of predefined indications for certain medications might have affected the consistency of treatment decisions over the study period. Although PSM is effective in balancing cases with measurable confounders, it does not eliminate unmeasured or residual confounding factors. Furthermore, the dosage and duration of the aforementioned medications as well as the interval between medication initiation and the onset of SIP were not evaluated in this study.

## 5. Conclusions

This first PSM-based study supported the finding that postnatal hydrocortisone exposure and combined inotrope use are independent risk factors for SIP in ELBW infants. Clinicians should closely monitor extremely preterm infants receiving hydrocortisone or combined inotropes for an elevated risk of SIP.

## Figures and Tables

**Table 1:** Summary of current studies aimed to identify pharmacologic treatment-related risk factors for SIP ^¶^.

Studies [Ref.]	Years	Methods	Population	SIP (n) ^§^		ANS	Mg	IND		SUR	HC	DEX	IND	IBU	ACE	CAF	MIL	DOP	DBU	EPI
Paquette [3]	2006	Matched C-C	BW <1500 g	16	Antenatal				Postnatal											
Attridge [4]	2006	Matched C-C	BW <1000 g	633																
Ahmad [5]	2008	Retro. cohort	BW <1000 g	13																
Wadhawan [6]	2013	Retro. cohort	BW <1000 g	280																
Rattray [7]	2014	Retro. cohort	BW <1000 g	24																
Shah [8]	2015	Retro. cohort	GA <32 week	178																
Shalabi [9]	2017	Retro. cohort	GA <28 week	148																
Stavel [10]	2017	Retro. cohort	BW <1000 g	101																
Rayyan [11]	2018	Matched C-C	GA <32 week	62																
Arnautovic [12]	2021	Matched C-C	GA <29 week	57																
Kandraju [13]	2021	Retro. cohort	BW <750 g	199																
Laptook [14]	2023	Retro. cohort	BW <1000 g	243																
Mantle [15]	2024	Retro. cohort	GA <30 week	23																
Thakkar [16]	2024	Retro. cohort	GA ≤24 week	379																
Our Study	2024	PSM C-C	BW <1000 g	28																

SIP: spontaneous intestinal perforation; C-C: case-control; Retro: retrospective; PSM: propensity score matching; BW: birthweight; GA: gestational age; ANS: antenatal steroid; Mg: MgSO_4_; IND: indomethacin; SUR: survanta; HC: hydrocortisone; DEX: dexamethasone; IBU: ibuprofen; ACE: acetaminophen; CAF: caffeine; MIL: milrinone; DOP: dopamine; DBU: dobutamine; EPI: epinephrine. ^¶^ Risk for SIP: increased (red column), no association (yellow column), not evaluated in the study (gray column). ^§^ indicates numbers of SIP cases in respective study.

**Table 2:** Demographic data for SIP cases and the control before (cohort) vs. after PSM (PSM-controls).

	SIP (*n* = 28)	Cohort (*n* = 829)	PSM-Controls (*n* = 84)	ASMD Before PSM	ASMD After PSM *
GA (weeks)	25.3 ± 2.1	26.2 ± 2.2	25.5 ± 1.7	0.4121	0.0735
Birthweight (g)	735 ± 167	771 ± 146	727 ± 167	0.2295	0.0479
Male (n (%))	19 (68%)	499 (60%)	55 (65%)	0.0097	0.0030
Birth year	2019.2 ± 2.6	2018.0 ± 2.8	2018.8 ± 2.3	0.4367	0.1549

SIP: spontaneous intestinal perforation; PSM: propensity score matching; ASMD: absolute standardized mean difference; GA: gestational age. * A smaller ASMD indicates minimal difference between two groups.

**Table 3:** Comparison between SIP cases and PSM-controls.

	SIP Cases (*n* = 28)	PSM-Controls (*n* = 84)	*p* Value
Demographics			
Gestational age (weeks)	25.3 ± 2.1	25.5 ± 1.7	0.720
Birthweight (g)	735 ± 167	727 ± 167	0.829
Male	19 (67.9%)	55 (65.5%)	0.835
Year of birth	2019.2 ± 2.6	2018.8 ± 2.3	0.464
Vaginal delivery	11 (39.3%)	30 (35.7%)	0.734
Apgar scores at 1 min	6 (5–7)	6 (5–7)	0.924
Apgar scores at 5 min	8 (8–9)	8 (8–9)	0.931
Apgar scores at 5 min <7	4 (14.3%)	19 (22.6%)	0.427
Maternal age (years)	32.6 ± 5.3	32.9 ± 5.2	0.827
Multiple birth	11 (39.3%)	21 (25.0%)	0.147
Maternal hypertension	5 (17.9%)	10 (11.9%)	0.423
Maternal diabetes	1 (3.6%)	6 (7.1%)	0.678
Chorioamnionitis	3 (10.7%)	13 (15.5%)	0.757
Maternal use of antibiotics	25 (89.3%)	70 (83.3%)	0.555
Conditions related to neonatal stress			
Chest compression	3 (10.7%)	8 (9.5%)	1.000
Mechanical ventilation	22 (78.6%)	70 (83.3%)	0.569
PPHN *requiring iNO*	1 (3.6%)	8 (9.5%)	0.446
IVH *any grade*	14 (50.0%)	33 (39.3%)	0.320
IVH *grade* ≥ 3	6 (21.4%)	11 (13.1%)	0.287
Initiation of feeding	7 (25.0%)	52 (61.9%)	0.001 *
Hypo-/hyperglycemia ^†^	6 (21.4%)	27 (32.1%)	0.281
Sepsis *culture-proven*	4 (14.3%)	5 (5.9%)	0.224
Pharmacologic regimens			
Antenatal steroid *any*	24 (85.7%)	79 (94.1%)	0.224
Betamethasone	21 (75.0%)	61 (72.6%)	0.805
Dexamethasone	4 (14.3%)	19 (22.6%)	0.427
Antenatal MgSO_4_	28 (100.0%)	77 (91.7%)	0.189
Antenatal indomethacin	22 (78.6%)	61 (72.6%)	0.533
Surfactant	19 (67.9%)	41 (48.8%)	0.090
Postnatal steroid *any*	7 (25.5%)	10 (11.9%)	0.940
Hydrocortisone	7 (25.0%)	8 (9.5%)	0.037 *
Dexamethasone	1 (3.6%)	3 (3.6%)	1.000
Ibuprofen *any*	12 (42.9%)	27 (32.1%)	0.303
Ibuprofen *IV*	9 (31.4%)	24 (28.6%)	0.720
Ibuprofen *oral*	3 (10.7%)	6 (7.1%)	0.688
Acetaminophen *oral*	3 (10.7%)	3 (3.6%)	0.164
Caffeine	2 (7.1%)	11 (13.1%)	0.512
Milrinone	6 (21.4%)	22 (26.2%)	0.614
Inotropic agents			
Dopamine	16 (57.1%)	37 (44.0%)	0.229
Dobutamine	11 (39.3%)	18 (21.4%)	0.081
Epinephrine	7 (25.0%)	6 (7.1%)	0.011 *
In combination			0.023 *
None	10 (35.7%)	43 (51.2%)	0.156
1 agent	7 (25.0%)	25 (29.8%)	0.629
2 agents	6 (21.4%)	14 (16.7%)	0.569
3 agents	5 (17.9%)	2 (2.4%)	0.020 *

SIP: spontaneous intestinal perforation; PSM: propensity score matching; PPHN: persistent pulmonary hypertension of newborn; iNO: inhaled nitric oxide. † hypoglycemia requiring additional dextrose effusion or hyperglycemia requiring insulin. * indicates statistical significance.

## Data Availability

All data generated or analyzed during this study are included in this article. Further enquiries can be directed to the corresponding author.

## Abbreviations

The following abbreviations are used in this manuscript:

SIP	spontaneous intestinal perforation
ELBW	extremely low birthweight
PSM	propensity score matching
ANS	antenatal steroids
